# Comparative quantification of *Leishmania infantum* in experimental phlebotomine sand fly infections using kDNA and single-copy *Meta-1* gene qPCR assays

**DOI:** 10.1186/s13071-025-07231-x

**Published:** 2026-01-24

**Authors:** Stefania Porcelli, Jorian Prudhomme, Jovana Sádlová, Barbora Bečvářová, Petr Volf, Jérôme Depaquit, Florence Robert-Gangneux

**Affiliations:** 1 Faculté de Médecine, Univ Rennes, Inserm, EHESPIrset (Institut de Recherche en Santè Environnement Travail), UMR S 1085, 2 avenue Prof Leon Bernard, 35043 Rennes Cedex, France; 2 Faculté de Pharmacie, Université de Reims Champagne Ardenne, UR ESCAPE-USC ANSES PETARD, Reims, France; 3 Department of Parasitology, Faculty of Science, Charles University, Prague, Czech Republic; 4 Pôle de Biologie Territoriale, Laboratoire de Parasitologie-Mycologie, Centre Hospitalo-Universitaire, Reims, France; 5 Laboratoire de Parasitologie-Mycologie, Centre Hospitalier Universitaire de Rennes, Rennes, France

**Keywords:** *Leishmania infantum*, Sand fly vectors, qPCR, kDNA, *Meta-1* gene, Parasite quantification

## Abstract

**Background:**

Leishmaniasis is a parasitic disease transmitted by female sand flies and caused by protozoan parasites of the genus *Leishmania*. Accurate quantification of parasite load within vectors is essential for understanding transmission dynamics and vector competence. This study compares two quantitative polymerase chain reaction (qPCR) methods for detecting and quantifying *Leishmania infantum* in three experimentally infected sand fly species (*Phlebotomus perniciosus*, *Phlebotomus argentipes*, and *Phlebotomus orientalis*).

**Methods:**

One method targets kinetoplast minicircle DNA, which offers high sensitivity but limited quantitative precision, while the other targets the single-copy *Meta-1* gene, providing more precise quantification but reduced sensitivity in low-level infections.

**Results:**

A positive correlation between the two molecular markers supports a combined approach to maximize both sensitivity and accuracy in surveillance and transmission studies. Following this methodological comparison, significant differences were observed in parasite proliferation among sand fly species and *L. infantum* strains, with *Ph. orientalis* confirmed as a highly competent vector for *Leishmania donovani* complex.

**Conclusions:**

Together, these findings highlight that combining high-sensitivity (kinetoplast DNA [kDNA]) and single-copy (*Meta-1*) targets enables both accurate and sensitive quantification of *Leishmania* infections in sand flies, improving the assessment of parasite–vector interactions.

**Graphical Abstract:**

**Figure:**
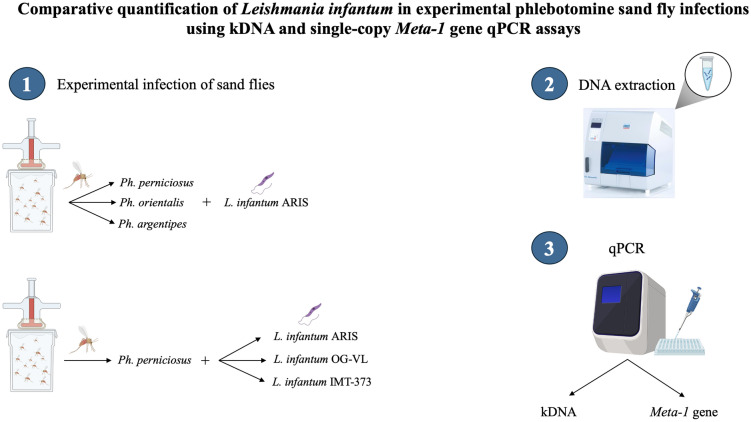

**Supplementary Information:**

The online version contains supplementary material available at 10.1186/s13071-025-07231-x.

## Background

Leishmaniasis is a parasitic disease spread through the bite of an infected female phlebotomine sand fly. It is caused by protozoan parasites belonging to the *Leishmania* genus, of which more than 30 species have been identified, approximately 20 of which are pathogenic to humans [1].

The disease manifests in several forms, most notably cutaneous, mucocutaneous, and visceral leishmaniases, each with different symptoms [2]. Visceral leishmaniasis, which is particularly prevalent in the Mediterranean region and is primarily caused by *Leishmania infantum*, is the most severe form and can be fatal; notably, disease outcome depends on several factors (e.g., host’s immune response and genetic background), and fatal cases may occur even with appropriate treatment.

Transmission occurs through the bite of infected female sand flies belonging to the Dipteran family Psychodidae. More than 1000 species of phlebotomine sand flies have been described to date [3]. Among the more than 1000 described species, approximately 98 have been recognized as confirmed or suspected vectors of human leishmaniasis, including 42 *Phlebotomus* species in the Old World and 56 *Lutzomyia* species in the New World, on the basis of their vector competence. This vector diversity is crucial for understanding the epidemiology and expanding distribution of leishmaniasis. This is influenced by an increase in breeding sites, changes in the availability of blood sources, the interruption of control activities, and environmental and climate changes [4].

The early detection of parasites within their vectors is crucial for evaluating transmission dynamics and vector competence, as well as for developing control interventions. Increasing knowledge on vector competency has been acquired through experimental infection of sand flies reproduced and fed in insectariums, which provide controlled conditions to investigate parasite development, survival, and differentiation within the midgut, as well as the complex interactions between parasite and vector tissues. Such experimental approaches are fundamental for elucidating mechanisms of vector competence, identifying barriers to parasite transmission, and understanding the impact of pathogens on their vectors [5–7]. Moreover, they allow the standardization of infection protocols that are not feasible in field studies, thereby providing critical insights into the dynamics of *Leishmania* transmission. The dissection of sand fly midguts several days after infection as well as microscopic examination to search for *Leishmania* parasites allow a rough estimation of the capacity of parasite multiplication in the vector. However, although quantitative polymerase chain reaction (qPCR) has been employed for quantifying experimental infections [8–11], its use is rarely intended for field-collected vectors.

Over the last decade, several studies have demonstrated that qPCR represents a highly sensitive and reproducible tool for detecting and quantifying *Leishmania* DNA in sand flies, offering a substantial improvement over traditional microscopy and culture-based methods. qPCR enables detection of very low parasite burdens, allows differentiation among species or strains through specific genetic targets, and provides quantitative data essential for understanding transmission dynamics [12, 13]. This molecular approach has been successfully used for entomological surveillance and for evaluating natural infection rates in field-collected vectors. For example, Jiménez et al. (2013) applied a kDNA-based qPCR to identify *L. infantum* in *Phlebotomus perniciosus* populations from human leishmaniasis foci in Spain, while Nzelu et al. (2025) developed a qPCR assay targeting the *sherp* gene to monitor *Leishmania* metacyclogenesis in sand flies, providing valuable information on transmission potential [14, 15].

In addition, qPCR could prove valuable for evaluating transmission dynamics in endemic areas. A kinetoplast DNA (kDNA) minicircle is widely used for diagnostic purposes in humans, due to its high sensitivity, which results from the presence of thousands of copies per parasite [16]. However, kDNA-based detection does not allow precise quantification of individual parasite genomes, as the number of repetitions can vary among parasite strains.

An alternative approach is to target the *Meta-1* gene, which, as Berberich et al. demonstrated, is present as a single copy in the genome of *L. infantum* and other *Leishmania* species [17]. The *Meta-1* gene encodes a conserved 29 kDa surface-associated protein involved in parasite differentiation and virulence. It is located in a stable, single-copy locus of chromosome 36 and is expressed predominantly in metacyclic promastigotes. Its conservation among both Old and New World species supports its use as a universal molecular marker [17, 18].

Southern blot analysis using multiple restriction enzymes has consistently produced a single *Meta-1*-specific fragment, confirming its single-copy status. This attribute enables direct and accurate quantification of parasite load by qPCR, with the number of *Meta-1* copies correlating with the number of parasites present in the sample. Furthermore, the *Meta-1* gene is highly conserved among different *Leishmania* species, enabling this molecular marker to be applied broadly across various strains and geographic regions [17].

In this study, our primary objective was to compare the performance of two molecular assays targeting the single-copy *Meta-1* gene and the highly repetitive kDNA minicircle target for the detection and quantification of *Leishmania* DNA in several experimentally infected sand fly species.

## Methods

### Sand flies and *Leishmania* parasite culture

Laboratory colonies of *Ph. perniciosus* (originally from Spain), *Phlebotomus argentipes* (originally from Turkey), and *Phlebotomus orientalis* (originally from Ethiopia) were maintained in the insectary of the Charles University in Prague under standard conditions (26 °C, fed on 50% sucrose and provided a photoperiod 14 h light/10 h dark) as previously described [19]. Parasites were cultured in M199 medium (Sigma) containing 10% heat-inactivated fetal calf serum (Gibco) supplemented with 2% sterile urine, 1% Basal Medium Eagle (BME) vitamins (Sigma) and 250 μg/mL amikacin (Medochemie LTD) at 23 °C. Three different *L. infantum* species were used: MCAN/IT/2003/ARIS (isolated from a dog in Italy), MHOM/TR/200/OG-VL (isolated from a human in Turkey), and MCAN/PT/2005/IMT373 (isolated from a dog in Portugal) [20].

The ARIS strain was used to prepare a calibrated standard. Briefly, 10 µL of parasite culture were collected after gentle homogenization of the culture, and promastigotes were counted by microscopy using a Kovac slide. Parasite concentration was adjusted to 10^8^/mL in a 1 mL volume. Serial dilutions to 1:10 were done until 1 parasite/mL in a volume of 200 µL and frozen at −20 °C until DNA extraction.

### Experimental infections of sand flies

Promastigotes from the logarithmic phase of cultivation were resuspended in heat-inactivated defibrinated sheep blood at a concentration of 3 × 10^6^ parasites per sand fly. Then, 120 female sand flies were fed through a chicken skin membrane, and engorged individuals were maintained under the same conditions as the colony until day 8 following the bloodmeal (PBM), when they were stored for DNA extraction.

### DNA extraction

DNA extraction was performed using the EZ1 DSP Virus Kit (Qiagen, France) on the EZ1 Advanced XL device. The EZ1 DSP Virus Kit used for DNA extraction was previously validated for *Leishmania* detection in phlebotomine sand flies, showing high yield and reproducibility across reference laboratories [21]. Each blood-fed female sand fly was transferred into a 2 mL screw-cap tube containing 1.4 mm MagNA Lyser Green Beads and 700 µL of Dulbecco’s phosphate-buffered saline (DPBS). Homogenization was carried out using the MagNA Lyser apparatus (Roche, France) for a single 30-s cycle at 3000 rpm. Following homogenization, 200 µL of the homogenate was combined with 200 µL of animal tissue lysis (ATL) buffer (Qiagen, France) and 20 µL of proteinase K, and incubated at 56 °C for 2 h. DNA was then extracted using 400 µL of the prepared lysate (200 µL homogenate + 200 µL ATL buffer) and eluted in a final volume of 90 µL. Extracted DNA was stored at −20 °C until use.

### Detection of kinetoplast DNA and *Meta-1* gene by qPCR

A real-time qPCR assay was used to detect and quantify *Leishmania* spp. DNA in sand fly samples, targeting both the kinetoplast minicircle DNA (kDNA) sequence and the *Meta-1* gene. Amplification of kDNA and was performed using the QuantStudio^™^ 5 system (QS5, Thermo Fisher, France). For kDNA, specific primers and a TaqMan probe were used, following the protocol described by (Coulson and Smith, 1990) (Table 1). Each 25 μL qPCR reaction mix included 5 μL of DNA sample, 12.5 μL of TaqMan Universal Master Mix 2X, and a final concentration of 0.5 μM of primers and 0.2 μM of probe. DNA was amplified using the following conditions: initial step at 95 °C for 10 min, followed by 45 cycles of 15 s at 95 °C and 1 min at 60 °C.

**Table 1:** Oligonucleotide primers used to detect *Leishmania* spp. kDNA and *Meta-1*

Target gene	Primer	Sequence (5′ → 3′)	Size (bp)	Reference
*Leishmania* kDNA	Leish F Leish R Leish P	5′-CTTTTCTGGTCCTCCGGGTAGG-3′ 5′-CCACCCGGCCCTATTTTACACCAA-3′ 5′-FAMTTTTCGCAGAACGCCCCTACCCGC3′-TAMRA	120 bp	[16]
*Leishmania* spp. *Meta-1*	Meta F Meta R	5′-CGCGGATCCATGGAGATGAAAAA CTTGCTT-3′ 5′-CCCAAGCTTCGCAGGAAC AAGCATGATGAT-3′	336 bp	[17]

Leish F, forward primer; Leish R, reverse primer; Leish P, TaqMan probe; Meta F, forward primer; Meta R, reverse primer. The gene was amplified using the SYBR Green method. Each 25 μL qPCR reaction consisted of 5 μL of DNA template, 12.5 μL of PowerUp^™^ SYBR Green Master Mix (Applied Biosystems, France), and *Meta-1*-specific primers (Table 1) at a final concentration of 0.5 μM. The thermal cycling conditions included an initial step at 95 °C for 10 min, followed by 45 cycles of 95 °C for 15 s and 60 °C for 1 min. Amplification specificity was verified through melting curve analysis.

The standard curve was established using a primary DNA extract containing a calibrated suspension of 10^6^ parasites/mL of the ARIS *L. infantum* strain, which was serially diluted five times at a 1:10 ratio. For each assay targeting both kDNA and the gene, a standard curve and a negative control were included. Each sample was run in duplicate. Quantification was performed using the standard curve, applying a threshold value of 0.02. The limit of detection was assessed by amplifying serial dilutions from the calibrated suspension until lack of target amplification.

### Statistical analysis

Parasite loads were compared using the Mann–Whitney test for independent groups and the Wilcoxon signed-rank test for paired data. Correlation between kDNA and quantification was evaluated using Spearman’s rank correlation. Statistical significance was set at *P* < 0.05. All statistical analyses and graph generation were carried out with GraphPad Prism version 10.

## Results

### Validation of the qPCR methods

The polymerase chain reaction (PCR) efficiency for the assay, calculated from the standard curve generated in duplicate, was 97.6% (Fig. S2B). The melting curve analysis allowed clear verification of amplification specificity (Fig. S1). The analytical detection limits were 10 parasites (or parasite equivalents)/mL for the kDNA assay (Fig. S2A) and 0.1 parasites/mL for the *Meta-1* assay (Fig. S2B).

### *Leishmania infantum *replication in *Ph. perniciosus* varies among strains

Quantification of *Leishmania* promastigote densities revealed significant variability among the three *L. infantum* strains in *Ph. perniciosus* at day 8 postinfection. Both kDNA and assays demonstrated that *L. infantum* ARIS and *L. infantum* OG-VL strains produced substantially higher parasite loads compared with *L. infantum* IMT-373. Moreover, the infection rates using assay showed that the OG-VL strain exhibited the highest sand fly infection prevalence, at 90% (27/30), followed by ARIS, at 82.14% (23/28), and IMT-373, at 64.52% (20/31) (Table 2).

**Table 2:** Infection rate of *Ph. perniciosus* individuals fed with three different *Leishmania* strains and analyzed by qPCR at day 8 of infection

*L. infantum* species	Number of positive sand flies/number tested with kDNA qPCR (%)	Number of positive sand flies/number tested with qPCR (%)
ARIS	27/28 (96.4)	23/28 (82.14)
OG-VL	29/30 (96.6)	27/30 (90)
IMT-373	30/31 (96.8)	20/31 (65.52)

**Fig. 1:**
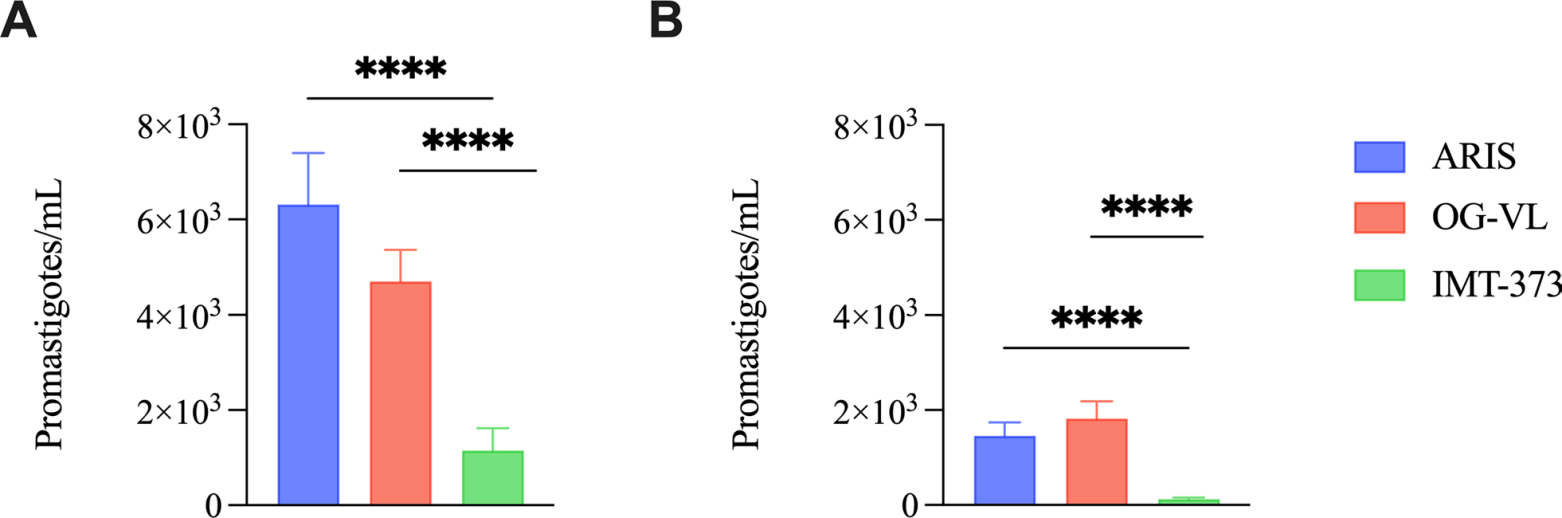
Quantification of different *L. infantum* strains (ARIS, OG-VL, IMT-373) in *Ph. perniciosus* on day 8 postinfection by qPCR. The figure shows the parasite loads quantified using kDNA (**A**) and qPCR assays (**B**). ^****^P < 0.0001 (Mann–Whitney *U* test). All values are presented as means ± standard error of the mean (SEM)

In the kDNA assay, mean promastigote concentrations in the *L. infantum* ARIS and *L. infantum* OG-VL groups reached approximately 6000 and 5000 parasites per sand fly, respectively, whereas *L. infantum* IMT-373 exhibited significantly lower levels, remaining close to 1000 parasites per sand fly (Mann–Whitney *U*-test, *U* = 35, *P* < 0.0001) (Fig. 1A). A similar pattern was observed in the assay, where *L. infantum* ARIS and *L. infantum* OG-VL maintained promastigote densities around 1500–2000 parasites per sand fly, while *L. infantum* IMT-373 levels were negligible (Mann–Whitney *U*-test, *U* = 41, *P* < 0.0001) (Fig. 1B). These results indicate that the *L. infantum* IMT-373 strain is considerably less efficient at proliferating in *Ph. perniciosus* in comparison to *L. infantum* ARIS and *L. infantum* OG-VL. Of note, the qPCR failed to detect 4/27 (14.8%) of *L. infantum* ARIS, 2/29 (6.9%) of *L. infantum* OG-VL, and 10/30 (33.3%) of *L. infantum* IMT-373 kDNA-positive samples (Table 2). None of the kDNA-negative samples were positive with the qPCR.

### Comparison of *L. infantum* ARIS infection in three *Phlebotomus* species

The second phase of the experiment compared three *Phlebotomus* species (*Ph. perniciosus*, *Ph. orientalis*, and *Ph. argentipes*) infected with the ARIS strain, and revealed significant differences in their ability to support *Leishmania* development. The results showed marked species-specific differences in infection rates: *Ph. orientalis* had a 100% infection rate (29/29), while *Ph. perniciosus* and *Ph. argentipes* had rates of 82.14% (23/28) and 73.30% (22/30), respectively, using assay (Table 3).

**Table 3:** Infection rate of *Leishmania infantum* ARIS at day 8 postinfection in three different *Phlebotomus* species

Sandfly species	Number of positive sand flies/number tested with kDNA qPCR (%)	Number of positive sand flies/number tested with qPCR (%)
*Ph. perniciosus*	27/28 (96.4)	23/28 (82.14)
*Ph. argentipes*	29/30 (96.7)	22/30 (73.30)
*Ph. orientalis*	29/29 (100)	29/29 (100)

**Fig. 2:**
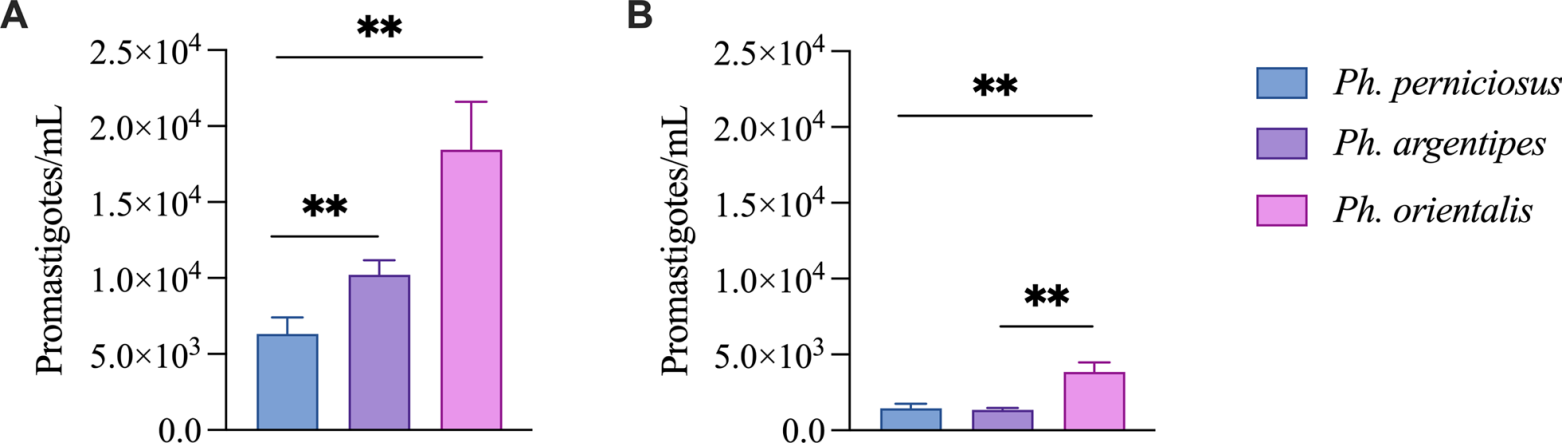
Quantification of *L. infantum* ARIS infection in three *Phlebotomus* species on day 8 postinfection by qPCR. The figure shows the parasite loads in three *Phlebotomus* species (*Ph. perniciosus*, *Ph orientalis*, and *Ph. argentipes*) using kDNA **A** and **B** qPCR assays. ^**^P < 0.01 (Mann–Whitney *U* test). All values are presented as means ± standard error of the mean (SEM)

Additionally, *Ph. orientalis* harbored significantly higher parasite loads than both *Ph. argentipes* and *Ph. perniciosus*. In the kDNA assay, mean promastigote densities in *Ph. orientalis* surpassed 20,000 parasites per sand fly, while *Ph. argentipes* and *Ph. perniciosus* reached approximately 10,000 and 6000 parasites per sand fly, respectively (Mann–Whitney *U* test: *U* = 135 and *U* = 166, respectively; *P* < 0.01) (Fig. 2A). The assay corroborated this trend: *Ph. orientalis* exceeded 5000 parasites per sand fly, whereas the other two species showed parasite densities below 2000 parasites per sand fly (Mann–Whitney *U* test: *U* = 152 and *U* = 169, respectively; *P* < 0.01) (Fig. 2B). Of note, the qPCR failed to detect 4/27 (14.8%) of *L. infantum* in *Ph. perniciosus*, and 7/29 (24.13%) in *P. argentipes*. None of the kDNA-negative samples were positive with the qPCR (Table 3).

**Fig. 3:**
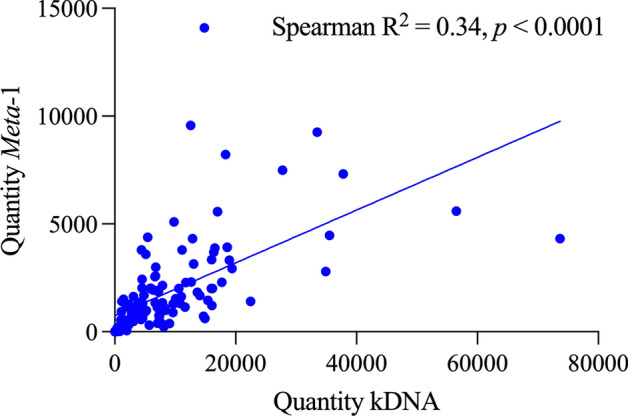
Correlation between *Leishmania* quantification by kDNA and qPCR in individual sand flies. Each point represents a single sample. A linear regression line is shown. Spearman’s correlation: *r*^*2*^ = 0.34, 95% CI 0.20–0.48, *P* < 0.0001 (*n* = 169)

Overall, parasite quantification was well correlated. Spearman’s correlation analysis revealed a significant positive correlation between the values obtained with the two markers (Fig. 3, Spearman’s *r* = 0.3455; 95% confidence interval [CI] 0.2009–0.4753; *P* < 0.0001; *n* = 169). To further evaluate agreement between the two quantification methods, a Wilcoxon signed-rank test for paired data was performed. This test detected a statistically significant difference between the quantities determined by kDNA and qPCR assays (Fig. 3; Wilcoxon signed-rank test: *P* < 0.0001; *n* = 169), with higher median values detected using the kDNA assay (median kDNA = 4.423; median = 1.316), consistent with data shown in Figs. 1 and 2, showing a three- to fourfold ratio between parasite loads calculated with kDNA and gene targets.

## Discussion

To evaluate the efficacy of multiplication of different *L. infantum* strains in *Ph. perniciosus*, we performed quantitative PCR analyses targeting the two DNA-based molecular targets, kDNA and *Meta-1*. Our first goal was to compare parasite loads across sand fly groups infected with three distinct *L. infantum* strains at day 8 postinfection. The data revealed significant variations in promastigote burden among the strains, whatever the gene target used for quantification, shedding light on the dynamics of parasite establishment and amplification specific to each strain within the vector. These observations may contribute to understanding variation in *L. infantum* infection levels among sand flies’ population in the Mediterranean basin.

The high sensitivity of kDNA-based qPCR for *Leishmania* detection is well documented, attributable to the presence of thousands of minicircle copies per parasite, estimated in the range of 5000–10,000 copies per cell (Mary et al. [16]; Nicolas et al. [22]). This confers an advantage for detecting low parasite burdens, but introduces challenges for quantitative interpretation due to potential variability in minicircle copy number among parasites and strains [23].

While kDNA qPCR provides very high sensitivity due to the presence of thousands of minicircle copies per parasite, variability in copy number among strains limits its quantitative accuracy [12, 13, 16]. Conversely, the single-copy target enables more accurate quantification [17, 18] but may fail to detect low parasite burdens, particularly during early infection stages. Therefore, kDNA is preferable for screening, whereas *Meta-1* is better suited for precise quantification in experimental infections. In addition to differences related to target copy number, the interpretation of results should take into consideration that the two assays rely on different qPCR chemistries, which could have an impact on the sensitivity comparison. Besides, while kDNA assay uses a TaqMan probe system, which provides high specificity through probe hybridization, SYBR Green chemistry requires careful melting curve analysis to confirm amplification specificity, which was done here. Overall, parasite quantification showed a good level of concordance. Spearman’s correlation analysis revealed a significant positive correlation between the values obtained with the two markers.

Our results presented a robust detection capacity of kDNA qPCR across different *L. infantum* strains and sand fly species also for low levels of infections [12, 13, 16]. Although useful for the diagnosis in humans, the significance of very low parasite loads in sand flies remains to be addressed. This could reflect the parasite’s inability to develop in the vector, as evidenced by occasional microscopic observations in which a few non-metacyclic promastigotes in the abdominal gut survived defecation but had no chance to infect the host, or in which only DNA remnants could be detected instead of viable parasites [24].

In contrast, the gene, described by Berberich et al. as a single-copy gene conserved across multiple *Leishmania* species [17], allowed for more precise genomic quantification of parasite load, with a 1:1 correlation between detected copies and parasites, but failed to detect low parasite load positivity with the kDNA qPCR. Our parallel evaluation revealed consistent findings in parasite loads across the three *L. infantum* strains (ARIS, OG-VL, and IMT-373) within *Ph. perniciosus*, with qPCR, providing lower but more stable quantification values correlating with expected parasite genome numbers. The smaller standard error of the mean (SEM) of parasite loads detected by each condition (Figs. 1, 2) using qPCR compared with kDNA qPCR partially reflects the stable copy number per genome. However, the qPCR failed to detect *Leishmania* in some kDNA-positive samples of ARIS (14.4%), of OG-VL (6.9%), and of IMT-373 (33.3%), highlighting its limited sensitivity, while all kDNA-negative samples remained negative.

The second goal of our study was to evaluate the added value of qPCR quantification of *Leishmania* in various *Phlebotomus* species. Similarly, our results revealed differential parasite burdens, with both *Meta-1*-based and kDNA-based quantification techniques, underscoring species-specific parasite replication dynamics according to the sand fly species [16, 17]. Notably, our results support the notion that *Ph. orientalis* is a highly competent vector for *Leishmania donovani* complex, capable of supporting parasite development to the infective stage [4, 25]. Overall, combining of kDNA and qPCR assays leverages the benefits of both markers: the high detection sensitivity of kDNA for screening purpose, and the precise parasite load quantification afforded by the single-copy gene. *Meta-1*, however, provides more stable measurements that fluctuate less artificially, but may fail to detect very low-level infections, thus cannot be a screening tool. However, it can remain an interesting option for quantification in experimental infections performed with known high concentrations, for example, when assessing the impact of infection on the vector. Multiple studies have confirmed that single-copy genes (e.g., *Meta-1*, DNA polymerase and HSP70) offer more reliable quantification of the true genomic parasite burden. Nevertheless, for early diagnosis and monitoring of latent infections, kDNA remains the preferred target due to its superior sensitivity [12, 16–18]. However, the epidemiological significance of the detection of very low parasite loads (or possibly residual DNA) is debatable. In that instance, qPCR might yield infection rates that would be more relevant for competency studies. However, it should be noted that even the sole quantification of the parasite is not decisive in determining vector competence; it must always be demonstrated that the parasite survives the vector’s defecation and develops a mature infection capable of transmission [26].

Beyond the genomic quantification performed in this study, future work should also explore molecular approaches capable of capturing the stage-specific expression patterns of *Meta-1*. In particular, developing a messenger RNA (mRNA)-based qPCR assay targeting transcripts or designing a specific TaqMan probe for this gene would provide valuable tools to investigate its transcriptional dynamics during metacyclogenesis. Such assays could help determine whether expression increases in tandem with the appearance of infective metacyclic promastigotes and thereby clarify its potential as a biomarker of mature, transmissible infections.

## Conclusions

This study provides a comparative evaluation of two qPCR approaches for detecting and quantifying *L. infantum* in experimentally infected sand flies. By directly comparing a highly sensitive kDNA-based assay with a single-copy *Meta-1* gene assay, we demonstrate that the two targets offer complementary strengths. The kDNA assay showed superior sensitivity, enabling detection of low-level infections, whereas the *Meta-1* assay provided more accurate and stable quantification of parasite burden, albeit with reduced sensitivity.

Using these tools, we identified significant differences in parasite proliferation among *L. infantum* strains in *Ph. perniciosus* and revealed marked vector-specific differences in susceptibility, with *Ph. orientalis* supporting substantially higher parasite loads and infection rates, confirming its high vector competence for the *L. donovani* complex.

Overall, our findings support the use of a dual-marker strategy that combines kDNA for sensitive screening with *Meta-1* for precise parasite quantification. This integrated approach improves the assessment of experimental sand fly infections and provides a more robust framework for studying parasite–vector interactions, vector competence, and transmission dynamics in leishmaniasis research.

## Supplementary Information

Supplementary material 1: Fig. S1. Melting curve analyses showing the melting temperature (Tm) peaks of the qPCR standard series prepared from a primary *L. infantum *ARIS DNA extract (10^6^ parasites/mL) with five 1:10 serial dilutions; replicates show a single sharp peak at ~85 °C, indicating a specific amplicon with no nonspecific products or primerdimers.

Supplementary material 2: Fig. S2. kDNA and *Meta-1 *qPCR standard curves generated from a primary *L. infantum *ARIS DNA extract at 10^6^ parasites/mL followed by five 1:10 serial dilutions. Panel A: kDNA standard curve. Panel B: *Meta-1 *standard curve.

## Data Availability

The authors affirm that all data supporting the findings of this study are freely and fully available without restrictions. All resources employed in the preparation of this article have been provided, and all analytical procedures are described in sufficient detail to enable independent assessment and verification of the reported results.
