# Supplementary figures and images for: Comparative quantification of Leishmania infantum in experimental phlebotomine sand fly infections using kDNA and single-copy Meta-1 gene qPCR assays
Source: Parasit Vectors. 2026 Jan 24;19:50. doi: 10.1186/s13071-025-07231-x (PMC12849146; doi:10.1186/s13071-025-07231-x)

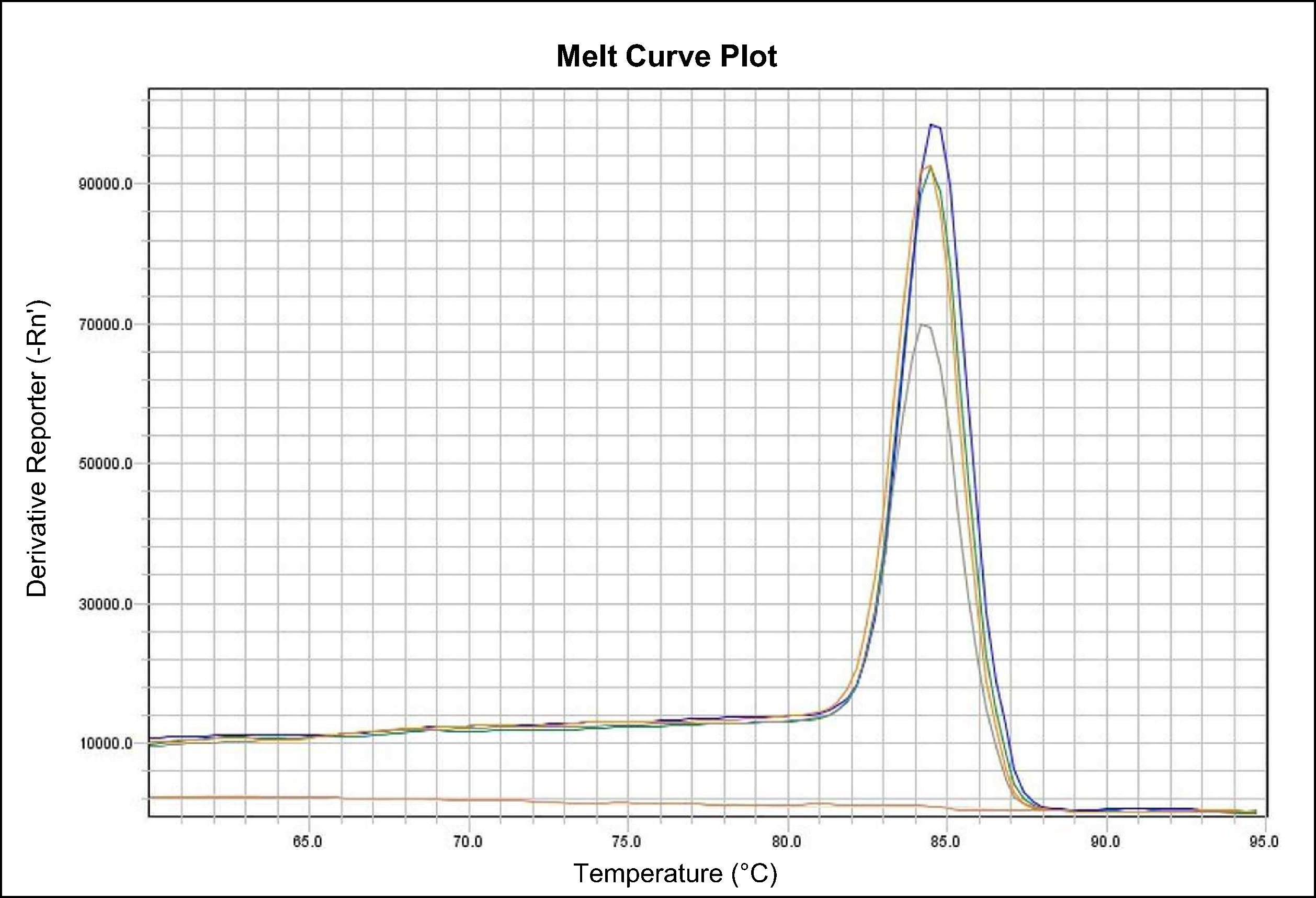

Supplement: Supplementary file 1 — Supplementary material 1: Fig. S1. Melting curve analyses showing the melting temperature (Tm) peaks of the qPCR standard series prepared from a primary L. infantum ARIS DNA extract (106 parasites/mL) with five 1:10 serial dilutions; replicates show a single sharp peak at ~85 °C, indicating a specific amplicon with no nonspecific products or primerdimers. [file 13071_2025_7231_MOESM1_ESM.tif]

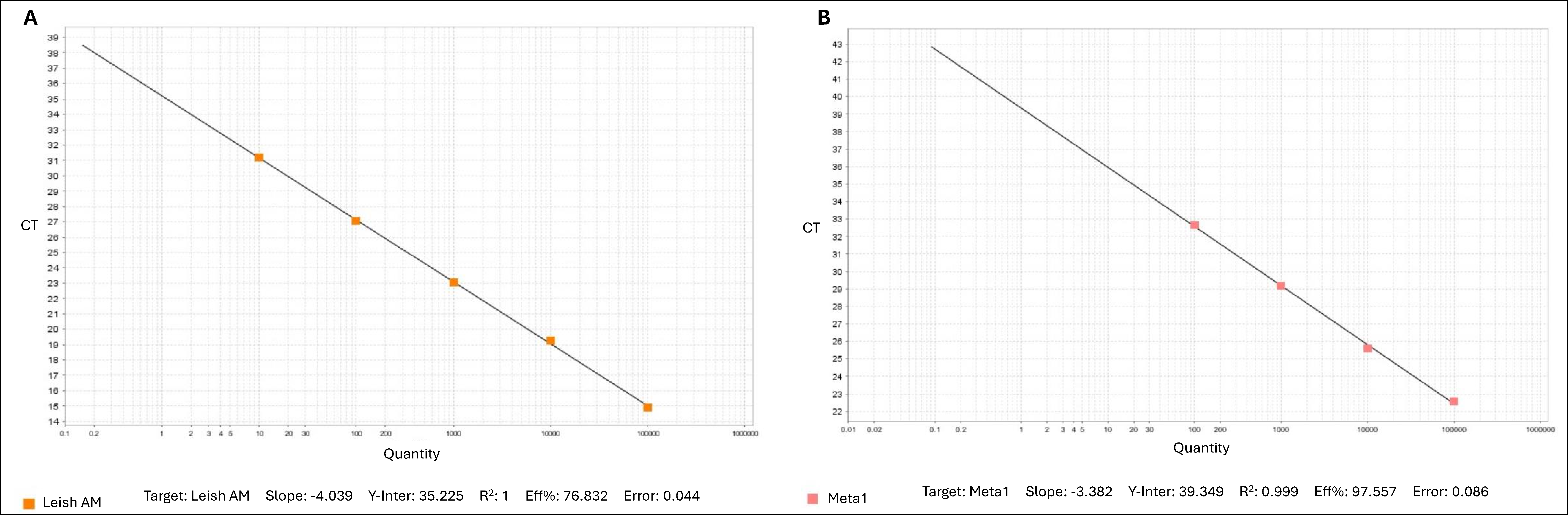

Supplement: Supplementary file 2 — Supplementary material 2: Fig. S2. kDNA and Meta-1 qPCR standard curves generated from a primary L. infantum ARIS DNA extract at 106 parasites/mL followed by five 1:10 serial dilutions. Panel A: kDNA standard curve. Panel B: Meta-1 standard curve. [file 13071_2025_7231_MOESM2_ESM.tif]
